# The Effects of Probiotic Supplementation on Anthropometric Growth and Gut Microbiota Composition in Patients With Prader-Willi Syndrome: A Randomized Double-Blinded Placebo-Controlled Trial

**DOI:** 10.3389/fnut.2021.587974

**Published:** 2021-02-19

**Authors:** Xue-Jun Kong, Guobin Wan, Ruiyi Tian, Siyu Liu, Kevin Liu, Cullen Clairmont, Xiaojing Lin, Xiaoying Zhang, Hannah Sherman, Junli Zhu, Yelan Wang, Michelle Fong, Alice Li, Bryan K. Wang, Jinghan Wang, Jun Liu, Zhehao Yu, Chen Shen, Xianghua Cui, Hanyu Cao, Ting Du, Xia Cao

**Affiliations:** ^1^Athinoula A. Martinos Center for Biomedical Imaging, Massachusetts General Hospital, Boston, MA, United States; ^2^Department of Medicine and Psychiatry, Beth Israel Deaconess Medical Center, Boston, MA, United States; ^3^Shenzhen Maternity and Child Healthcare Hospital, Shenzhen, China; ^4^PWS Care & Support Center, Wenzhou, China; ^5^Hangzhou Seventh Hospital, Hangzhou, China; ^6^Yale University, New Haven, CT, United States; ^7^Bentley University, Waltham, MA, United States; ^8^Brandeis University, Waltham, MA, United States; ^9^New York University, New York, NY, United States; ^10^Harvard Medical School, Boston, MA, United States; ^11^Second Affiliated Hospital of Kunming Medical University, Kunming, China

**Keywords:** Prader-Willi syndrome, microbiome, probiotics, *Bifidobacterium animalis* subsp *lactis*, obesity, inflammation, height, microbiota (microorganism)

## Abstract

**Background:** Prader-Willi Syndrome (PWS) is a rare genetic disorder associated with developmental delay, obesity, and neuropsychiatric comorbidities. *Bifidobacterium animalis* subsp. *lactis* has demonstrated anti-obesity and anti-inflammatory effects in previous studies.

**Aim:** To evaluate the effects of *Bifidobacterium animalis* subsp. *lactis* probiotics supplementation on anthropometric growth, behavioral symptoms, and gut microbiome composition in patients with PWS.

**Methods:** Ethical Approval was issued by the Internal Review Board (IRB) of the Second Affiliated Hospital of Kunming Medical University (Review-YJ-2016-06). We conducted a 12-week, randomized, double-blind, placebo-controlled trial in 68 patients with Prader-Willi syndrome aged 11 months−16 years (mean = 4.2 years old) who were randomly assigned to receive daily *B. lactis*-11 probiotics (6 × 10^10^ CFUs) or a placebo sachet. Weight, height, ASQ-3, ABC, SRS-2, and CGI-I were compared between the two groups at baseline and at 6 and 12 weeks into treatment. Gut microbiome data were analyzed with the QIIME 2 software package, and functional gene analysis was conducted with PICRUSt-2.

**Results:** We found a significant increase in height (mean difference = 2.68 cm, *P* < 0.05) and improvement in CGI-I (*P* < 0.05) in the probiotics group compared to the placebo group. No significant change in weight or psychological measures were observed. Probiotic treatment altered the microbiome composition to favor weight loss and gut health and increased the abundance of antioxidant production-related genes.

**Conclusions:** The findings suggest a novel therapeutic potential for *Bifidobacterium animalis* subsp. *lactis* probiotics in Prader-Willi syndrome patients, although further investigation is warranted.

## Introduction

Prader-Willi Syndrome (PWS) is a rare genetic imprinting disorder with an estimated prevalence of 1/10,000–1/30,000 (1). Three mechanisms cause this genetic disorder: deletion (DEL) of the 15q11.2-q13 region from the paternal chromosome, maternal uniparental disomy (UPD) from the mother, and imprinting defect (2). PWS is characterized by severe hypotonia and feeding difficulties in early infancy, and subsequent hyperphagia and morbid obesity starting during early childhood (1). PWS patients also typically experience generalized neurodevelopmental delays and numerous neuropsychiatric comorbidities (3).

Gut microbiota has been implicated in the etiology of obesity and associated comorbidities in PWS subjects (4). The gut microbiome from normal-weight patients has been found to have higher phylogenetic diversity than that from overweight and obese patients (5). In previous studies, people with diet-induced obesity and obese PWS patients were found to have similar gut dysbiosis (5). Dysbiotic gut microbiota transplanted from PWS patients to rats caused impacted expression of GLP-1 and decreased insulin-receptor signaling 2 weeks prior to an increase in body fat composition, indicating that the gut microbiome dysbiosis may play a role in the development of obesity (6). Administration of probiotics has shown improvement of metabolic disturbance and normalization of gut microbiome composition in diet-induced obese mice and in a randomized controlled trial of weight management in overweight adults (7, 8). Microbiome dysbiosis is not only related to obesity but also closely associated with neuropsychiatric conditions (9). Altered microbiome could serve as a biomarker for diagnosis and subtyping of Autism Spectrum Disorder (ASD) (10). Probiotics treatments have been broadly used to help people with neuropsychiatric conditions (11, 12).

*Bifidobacterium animalis* subsp. *lactis* (*B. lactis*) is a rod-shaped, anaerobic bacteria that can be found in the gastrointestinal tract of most mammals, including humans (13). Many strains of *B. lactis* are considered to be health-promoting and are commonly formulated into fermented dairy foods. Anti-obesity effects have been linked to the administration of some strains of *B. lactis*, such as A6, CECT 8145, Bf141, B420, and BB-12, mostly in animals (14–19). Anti-inflammatory effects of some strains of *B. lactis*, such as HN019 and BB-12, have also been reported in recent years (20, 21). Furthermore, a recent publication reported that *B.lactis* BPL1 improve abdominal adiposity and insulin sensitivity in children and adolescents with PWS (22).

In this study, we conducted a randomized, double-blind, placebo-controlled trial to test our hypothesis that probiotics consumption has beneficial effects on obesity, mental health, and inflammation associated with gut microbiome changes in PWS. We enrolled a cohort of PWS patients to evaluate the efficacy of a *B. lactis* strain (BL-11) on their weights, heights, psychological measurements, and gut microbiome compositions and functions relative to placebo controls. In addition to potentially supporting a new intervention for patients with PWS, the microbiome composition data collected from this study may shed light on the underlying mechanisms of PWS pathology and the gut-brain axis.

## Materials and Methods

### Study Design

**Figure 1:**
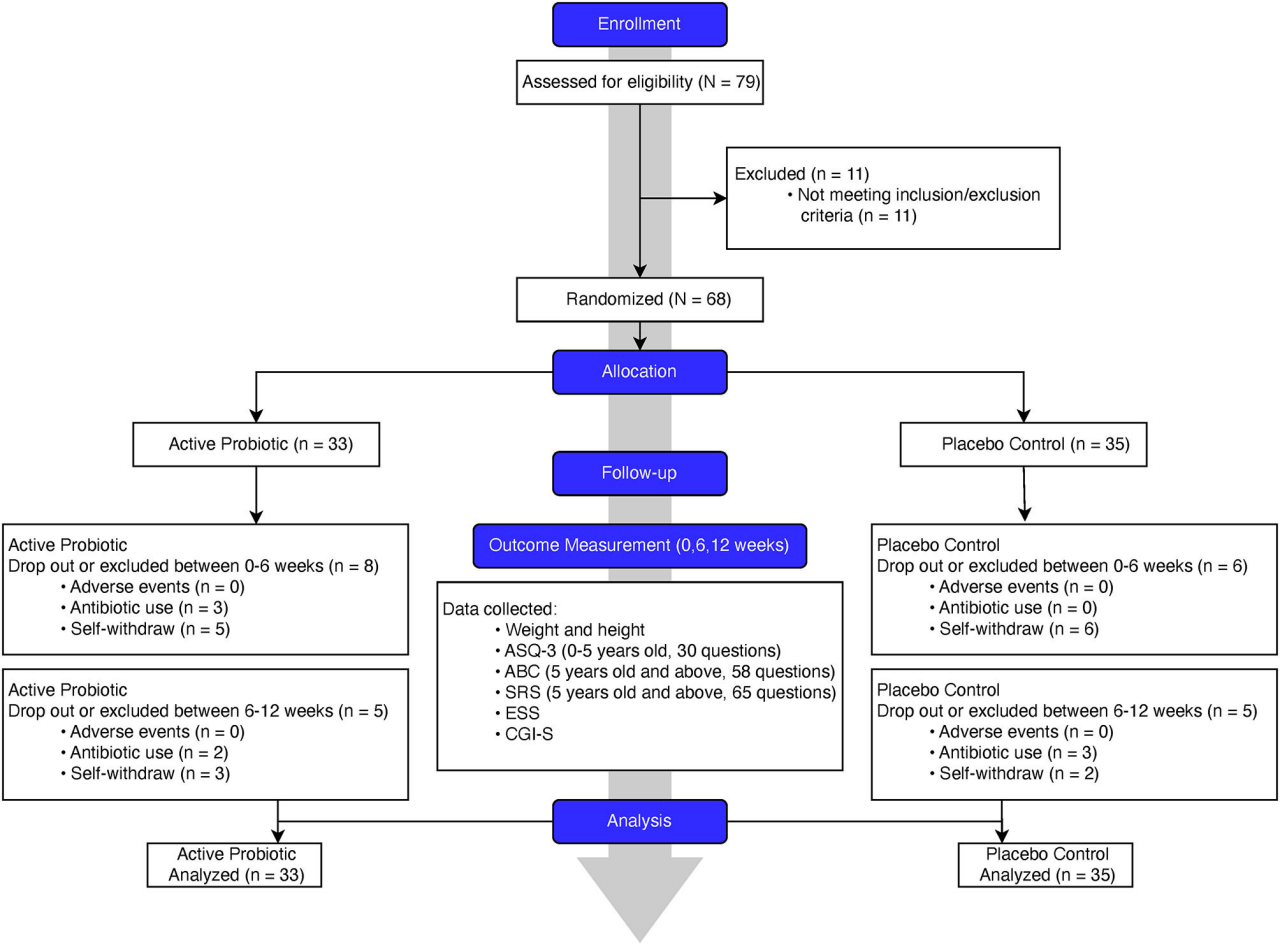
Flowchart of study conduct and procedure.

We designed and conducted a randomized, double-blinded, placebo-controlled clinical trial (flowchart, Figure 1). In this trial, we randomly assigned the eligible PWS participants, with a 1:1 ratio, to either the probiotics or placebo group. We hypothesize that a 12-week treatment period is sufficient for probiotics supplementation to induce detectable changes. To achieve a statistical power of 80% for primary outcomes with a large effect size of 0.8 (Cohen's *d*) assumed, a total of 52 participants (26 in each arm) were required.

### Ethical Considerations

Ethical Approval was issued by the Internal Review Board (IRB) of the Second Affiliated Hospital of Kunming Medical University (Review-YJ-2016-06). Clinical Trial of Probiotics was registered at the Chinese Clinical Trial Registry (ChiCTR), with a number ChiCTR1900022646. Signed informed consent was obtained from the parents or legal guardians of the subjects according to the IRB requirements. The study was conducted in accordance with the Declaration of Helsinki.

### Participants

We enrolled 68 subjects aged 50.5 ± 37.3 months (69.1% male, 30.9% female) with genetically confirmed diagnosis of Prader-Willi syndrome. Study participants were recruited through the PWS Care & Support Center, located in Zhejiang, China. Participants were included if they met the following criteria: they had been genetically confirmed to have PWS; had not been on any forms of probiotics for at least 4 weeks; had stable medications for at least 4 weeks; had no planned changes in medications or psychosocial interventions during the trial; had a willingness to provide stool samples in a timely manner; and had a willingness to collaborate with interviews and study procedures. Potential participants were excluded if they had other known genetic disorders, or if they were pregnant or breast-feeding before the study.

### Randomization and Blinding

Randomization and allocation concealment were performed by a statistician who was not part of the research team. Randomization sampling numbers were electronically generated for each de-identified subject. Coded probiotics and placebo of identical appearance were prepared by the Beijing Huayuan Academy of Biotechnology to ensure allocation concealment. Both the participants and the research staff/investigators who collected and analyzed the outcome data were blinded to treatment status. Blinding was also maintained by making the probiotics package appear identical to the placebo sachet.

### Intervention

Probiotics BL-11 (Beijing Huayuan Academy of Biotechnology) was used in the study in the format of a sachet containing the probiotic BL-11 in powder form. Each sachet of probiotics supplement contained 3 × 10^10^ colony forming units (CFUs). The placebo was maltodextrin in the sachet with similar color, flavor, and taste as the probiotic sachets. Subjects received one sachet twice a day of either probiotics or a placebo for a duration of 12 weeks and were instructed to consume the sachet contents orally with water.

#### Primary Outcomes

1. Weight and height measurements were obtained by parents using standard scales and collected by the research staff. Weight, height, and BMI were converted to z-score using age growth references provided by WHO (22).
2. Psychological measurements
  1. Ages and Stages Questionnaires, 3rd Edition (ASQ-3) (23). ASQ-3 is one of the most widely available development screening tools for young children. The ASQ-3 has five domains: communication, gross motor, fine motor, problem-solving, and personal-social. Total scores were calculated. We interviewed all subjects younger than 5 years old.
  2. Aberrant Behavior Checklist (ABC) (24). ABC is a 58-item behavior rating scale used to measure behavior problems across five subscales: irritability, lethargy/social withdrawal, stereotypic behavior, hyperactivity/non-compliance, and inappropriate speech. Total scores were calculated. We interviewed all subjects older than 5 years old.
  3. Social Responsiveness Scale (SRS)(25). SRS consists of 65 items used for quantitative assessment of the severity of social behaviors. Total scores were calculated. We interviewed all subjects older than 5 years old.
  4. Restricted and repetitive behaviors (RRB) is based on a 4-point scale (0–3) adopted from the Gilliam Autism Rating Scale, Third Edition (GARS-3) (26). Total scores were calculated. We interviewed all subjects older than 3 years old.

#### Secondary Outcomes

1. Fecal microbiome
  1. Sample Handling and Collection
    Stool samples were collected at three study timepoints: prior to intervention (0-weeks), 6 and 12-weeks. Sample collection was performed with DNA/RNA shield fecal collection tubes (Zymo, Cat#R1101) containing 1 mL preservation solution and were transported to the laboratory by ice bags and then frozen at −80°C. TIANmap stool DNA kit was used to extract DNA (TIANGEN, Cat#DP328) according to the manufacturer's instructions, and DNA samples were carefully quantified with a Nanodrop Spectrophotometer. A260/A280 ratios were also measured to confirm high-purity DNA yield. DNA samples were frozen at −20°C until use.
  2. 16S rRNA Gene Amplicon Sequencing
    The 16S rRNA V3-V4 library was constructed by two rounds of PCR with the following primers: 341F:5′TCGTCGGCAGCGTCAGATGTGTATAAGAGACAGCCTACGGGAGGCAGCAGCCTACGGGNBGCASCAG3′ and 805R:5′GTCTCGTGGGCTCGGAGATGTGTATAAGAGACAGTGACTACNVGGGTATCTAATCC3′ via reaction procedure (95°C for 2 min, followed by 25 cycles at 95°C for 30 s, 55°C for 30 s, and 72°C for 30 s, and a final extension at 72°C for 5 min). PCR products were purified with 1x KAPA AMPure beads (KAPA, Cat#KK8002). Then, products were put through a second PCR reaction procedure (95°C for 2 min, followed by eight cycles at 95°C for 30 s, 55°C for 30 s, and 72°C for 30 s, and a final extension at 72°C for 5 min). PCR products were purified with 1x KAPA AMPure beads and analyzed using a Bioanalyzer DNA kit, followed by quantification with real-time PCR. DNA libraries were pooled and sequenced on Illumina MiSeq (Illumina; CA) using a 2 × 250 bp paired-end protocol with overlapping reads.
2. Clinical Global Impression (CGI) was developed for use in clinical trials to provide a brief, stand-alone assessment of the clinician's view of the patient's global functioning prior to and after initiating a study medication. The CGI comprises two companion one-item measures evaluating the following: (a) severity of psychopathology from 1 to 7 (CGI-S) and (b) change from the initiation of treatment on a similar seven-point scale (CGI-I)^31^.
3. GI symptoms were assessed based on the total number of existing GI symptoms at baseline, including constipation, diarrhea, abdominal pain, excessive flatulence, bloody stool, nausea, difficulty swallowing, poor appetite, indigestion, and acid reflux.

### Statistical Analysis

All raw data were recorded and processed in Microsoft Excel 2007 and R. The presentation of data follows the CONSORT recommendations for reporting results of Randomized Clinical Trials (RCTs). Statistical procedures were carried out using α = 0.05 as the significance level.

We applied the Wilcoxon rank-sum test to explore the intergroup differences in the z-scores of weight, height, total scores and sub-scores of ASQ-3, ABC, and SRS at baseline, per-subject changes from 0 to 6 weeks, and per-subject changes from 6 to 12 weeks. Linear mixed models were also used to account for repeated measures.

Due to having several primary outcomes, false discovery rate (FDR) was used to adjust for multiple comparisons. Secondary outcomes were analyzed using similar methods as that of primary outcomes. In addition, linear regression was performed to check for correlations between clinical indices and microbiome compositions.

### Microbiome Data Processing and Analysis

The sequencing reads were filtered using the QIIME2 (v2019.10) based on quality scores (27). Deblur was used to denoise with default parameters and obtain an abundance table of samples by amplicon sequence variants (ASVs) (28).

Alpha diversities were calculated with QIIME2. Bray-Curtis distance was used to characterize microbiome beta diversity. Taxonomies for ASVs were assigned using the sklearn-based taxonomy classifier trained on the sequences at 99% similarity level from Greengenes v13.8. Significant differences in the relative abundance of microbial phyla, genera, and alpha diversity between placebo and probiotics groups were identified by Kruskal–Wallis tests. A false discovery rate (FDR) based on the Benjamini–Hochberg (BH) adjustment was applied for multiple comparisons (29).

PICRUSt2 was used to infer microbial functional content based on ASVs' abundant tables and then produced the Kyoto Encyclopedia of Genes and Genomes (KEGG) orthologs (KO), Enzyme Classification numbers, and pathway abundance table (30, 31). The differential analyses were performed on the fold ratios between probiotics and placebo groups with a permutation-based non-parametric test, and the top differential features were rendered and plotted with Calour (32). All raw data from 16s rRNA Illumina amplicon sequencing have been deposited in The National Center for Biotechnology Information (NCBI) Sequence Read Archive (SRA, PRJNA643297).

## Results

### Demographic Features of PWS Participants

A total of 68 subjects with genetically confirmed diagnosis of Prader-Willi syndrome were enrolled. Of which, 33 subjects aged 47.2 ± 32.4 months were randomized to receive active probiotic, BB-11, while 35 subjects aged 53.9 ± 42.0 months were randomized to receive placebo. Groupwise comparisons of baseline age and gender distributions did not indicate any significant differences (*P* > 0.05). Detailed demographic characteristics and co-morbid GI symptoms of the enrolled participants are summarized in Table 1. The overall severity presented by CGI-S scores at baseline compared between groups is presented in Supplementary Figure 1. No group differences were observed (*P* > 0.05). 47.5% of subjects display one or more GI symptoms within the study population.

**Table 1:** Demographic features and co-morbid GI symptoms of participants.

		**Probiotic group**	**Placebo group**	*P*-value^*^
Age [months, *N* (mean ± SD)]	All subjects	33 (47.2 ± 32.4)	35 (53.9 ± 42.0)	0.47
	> 5 years	11 (90.3 ± 26.1)	11 (106.6 ± 32.9)	0.27
	≤ 5 years	22 (28.9 ± 12.3)	24 (31.1 ± 14.5)	0.12
Sex [*N* (%)]	Male	22 (67%)	25 (71%)	0.73
	Female	11 (33%)	10 (29%)	
Genotype [*N* (%)]	Deletion	20 (61%)	22 (63%)	0.70
	Disomy	2 (6%)	3 (9%)	
	Other/Unknown	11 (33%)	10 (29%)	
Weight (kg, mean ± SD)		20.2 ± 15.0	24.2 ± 17.2	0.32
Height (cm, mean ± SD)		99.2 ± 20.0	104 ± 22.8	0.36
BMI (mean ± SD)		18.5 ± 6.4	19.8 ± 6.6	0.45
GI Symptoms (has ≥ 1)		0.87 ± 1.63	1.39 ± 1.75	0.24

* *Chi-square test was performed on sex and genotype, for which the p-values were >0.05 across the groups. T-test was performed on age, weight, height, BMI, and GI symptoms for which P-values were non-significant at α = 0.05 across the groups*.

No serious or severe adverse events were observed. All the observed adverse events and major causes of drop out are listed in Supplementary Table 1. There was no significant difference found between the two groups (*P* > 0.05).

### Effects of Probiotics on Weight, Height, Psychological Measurements, and CGI-I

**Figure 2:**
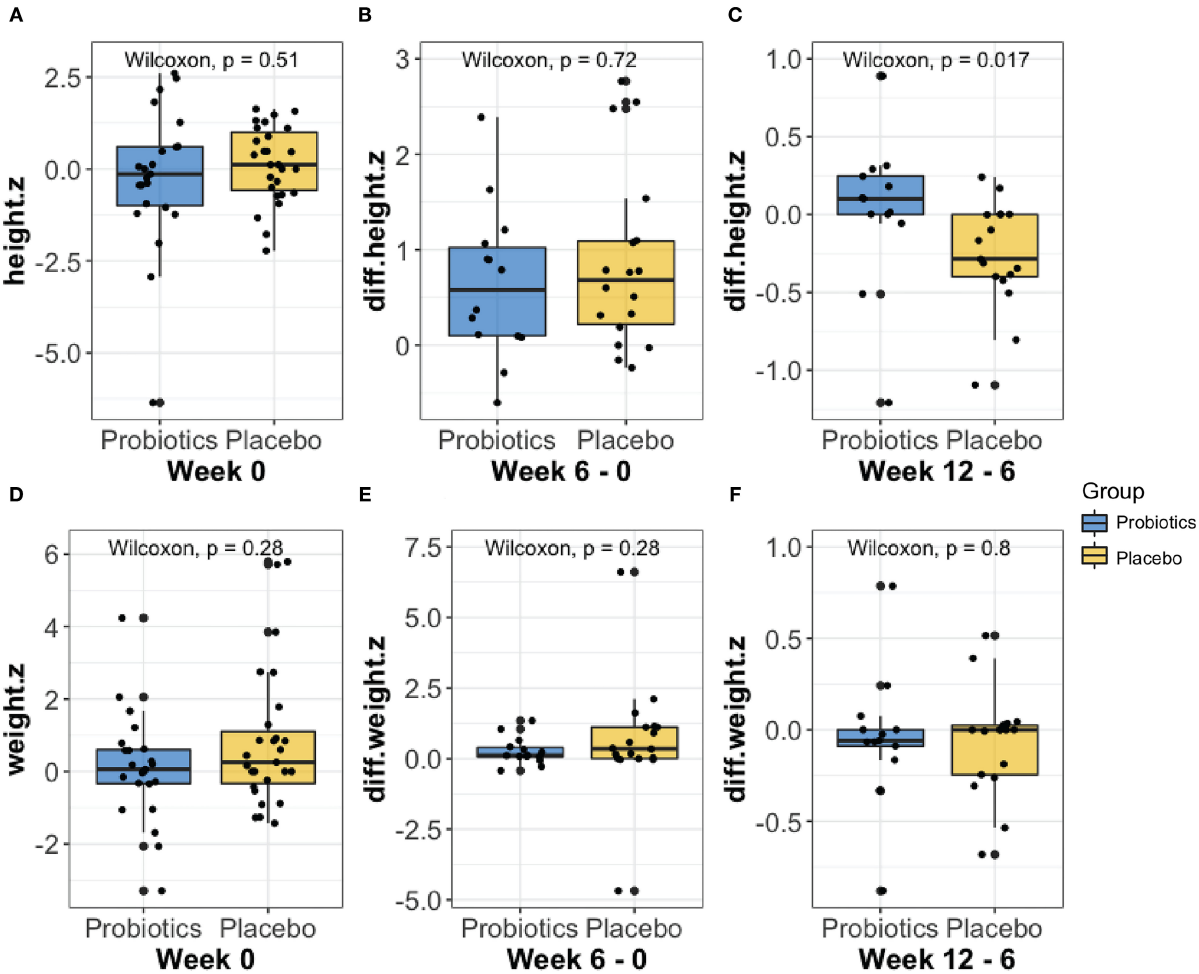
Comparison of the height **(A–C)** and weight **(D–F)** z-score changes at baseline, from week 0 to 6, and from week 6 to 12 between probiotic groups (blue) and placebo (yellow) using Wilcoxon rank-sum test. As shown in **(C)**, the probiotics group had a significantly greater height (*P* < 0.05) increase than the placebo group from week 6 to 12.

Anthropometric measures were collected and analyzed throughout the treatment course. No significant groupwise differences in mean height (z-score) were observed at baseline (week 0, Figure 2A). Furthermore, we did not observe any significant groupwise difference in the mean change in height (z-score) between 0- to 6-weeks (Figure 2B). The height increase from 6 to 12 weeks was significantly greater in the probiotics group than the placebo group (mean difference = 2.58 cm, *P* < 0.05, Figure 2C). No significant changes in weight over time were observed in either group (Figures 2D–F).

**Figure 3:**
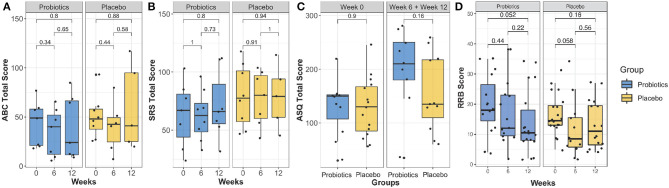
Comparison of the ABC total score **(A)**, SRS-2 total score **(B)**, ASQ-3 total score **(C)** and RRB score **(D)** over the intervention course between probiotics group (blue) and placebo group (brown). There was no group significance found (*P* > 0.05).

Results obtained from psychological measurements, including the ASQ-3, ABC, SRS, and RRB, are shown in Figure 3. No significant difference was found with the linear mixed effect model (*P* > 0.05) for ASQ-3, ABC, SRS, and RRB scores.

**Figure 4:**
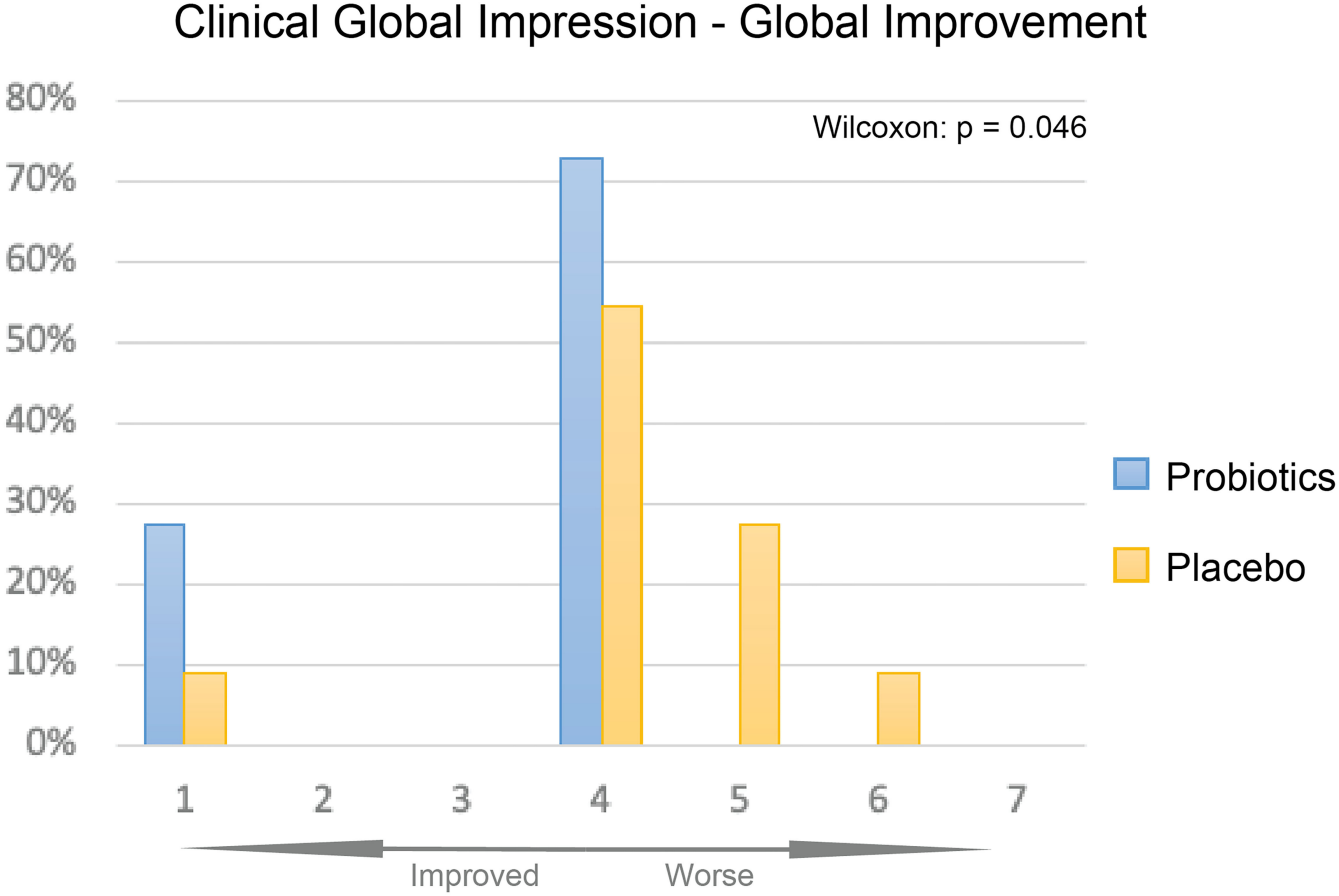
CGI-I of probiotics and placebo at 12 weeks. Percentage of participants given each improvement level was displayed as bar plot, probiotics group (blue) had overall significantly better improvement than the placebo group (yellow, *P* < 0.05).

The overall improvement of symptoms during the treatment course was measured using the CGI-I scale. We observed significantly greater symptom improvement in the probiotics group compared to the placebo group (Figure 4, *P* < 0.05).

### Changes in Microbiome Composition and Function With Probiotics Intervention

**Figure 5:**
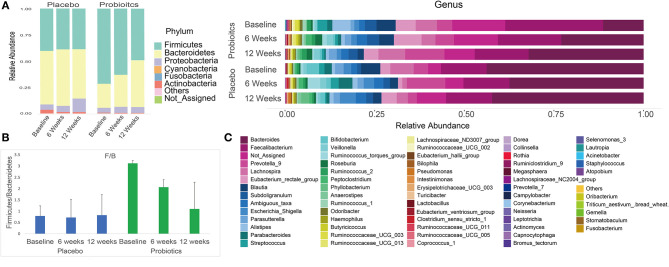
Summary of phylum and genus level gut microbiota relative abundances in both probiotics and placebo group subjects at baseline, 6 and 12 weeks. **(A)** Phylum level gut microbiota relative abundance per group at each study visit. **(B)** Firmicutes/Bacteroidetes ratio per group at each study visit. **(C)** Genus level gut microbiota relative abundance per group at each study visit.

After sequencing, we obtained a total of 3,088,722 raw reads and an average of 49,818 reads per sample (range = 29,329–119,440 reads). Overall phylum and genus level variations in gut microbiota composition over the intervention course are shown in Figure 5 for both probiotics and placebo groups.

**Figure 6:**
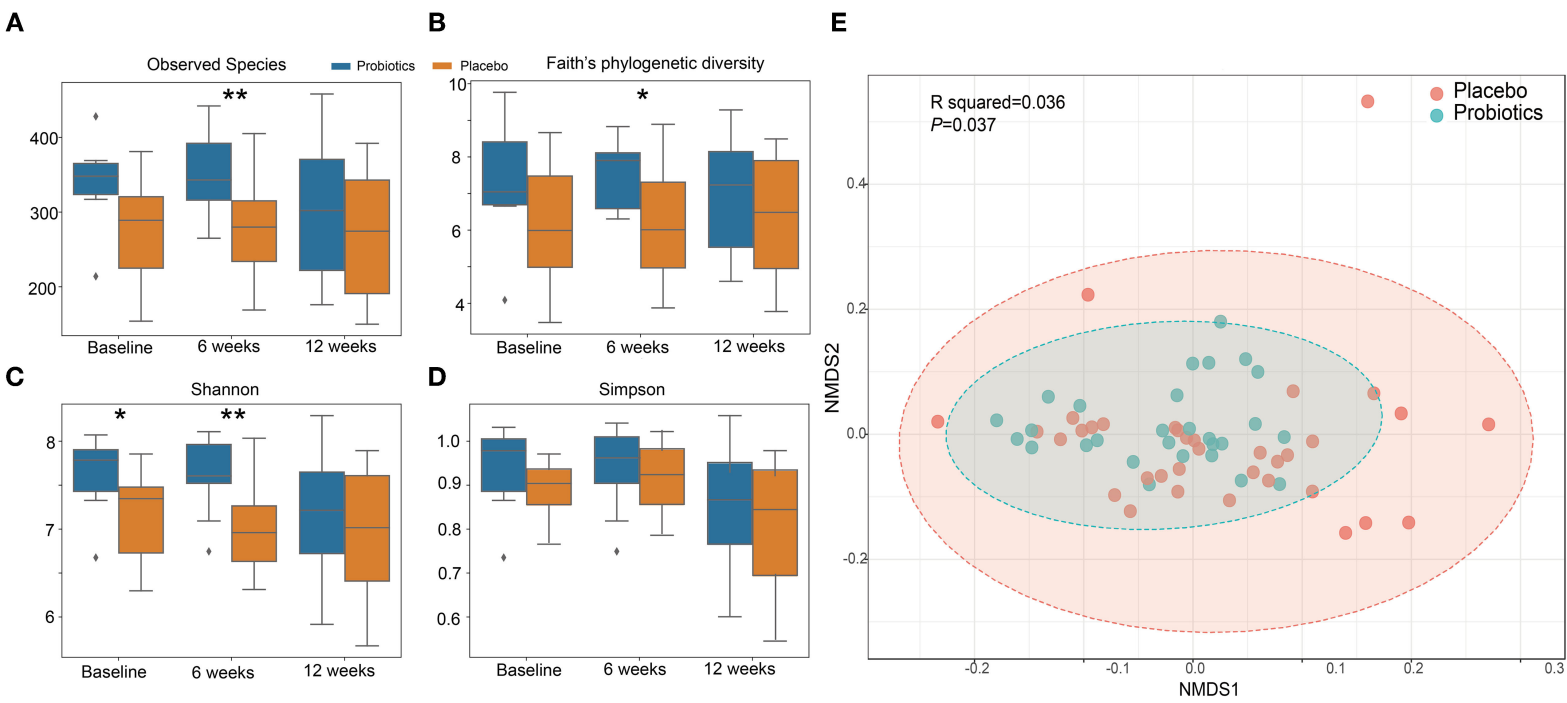
α and β diversity index changes from probiotics intervention. **(A)**: observed species index; **(B)**: faith's phylogenetic diversity; **(C)**: Shannon index; **(D)**: Simpson index. *P < 0.05; **P < 0.01, via *t*-test. **(E)**: β diversity with Non-metric multidimensional scaling (NMDS) score plots of gut microbial data based on a Bray–Curtis dissimilarity matrix. Placebo (red dots) and probiotics (blue dots).

α diversity slightly but significantly increased in the probiotics group compared with the placebo group after 6 weeks (Figures 6A–D). β-diversity, analyzed by a permutational multivariate ANOVA (PERMANOVA), showed a significant separation with probiotics treatment (F-statistic = 2.2526; *R*^2^ = 0.035613; *P* < 0.05, NMDS Stress = 0.19048, Figure 6E).

**Figure 7:**
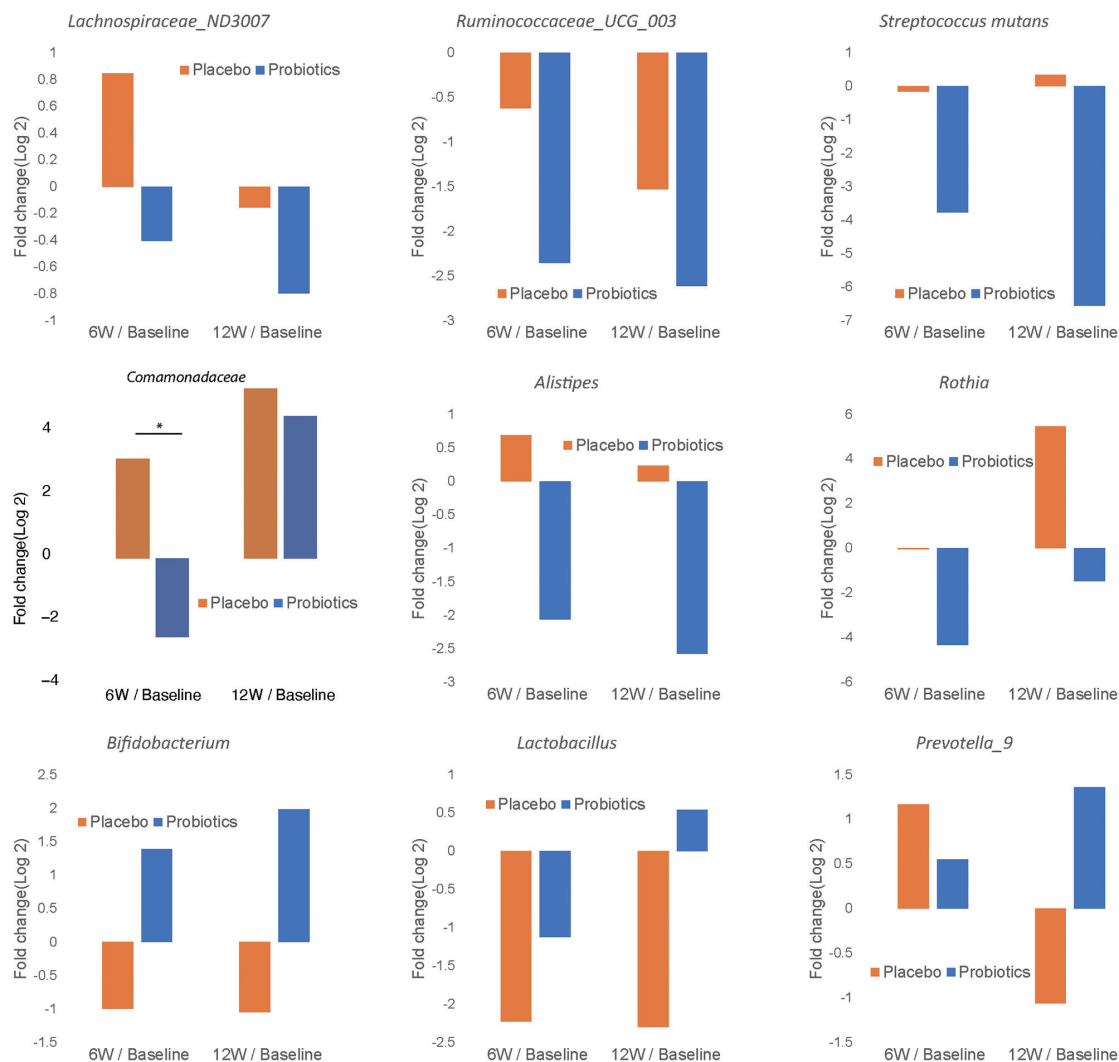
Fold change of relative abundance at genus/species level over the course of intervention for the probiotics group (blue) and placebo (orange). Each bar represents the log 2 transferred relative change of gut microbial abundance of 6 and 12 weeks compared with the baseline. Significant differences are marked with * to indicate *P* < 0.05.

In order to characterize the change in abundance of potentially clinically significant bacteria over the intervention course, we presented the fold changes of several selected bacterial genera and families in Figure 7. The relative abundances of *Lachnospiraceae* ND3007, *Ruminococcaceae* UCG-003, *Streptococcus mutans, Comamonadaceae, Alistipes*, and *Rothia* showed decreasing trends from baseline levels in the probiotics group at both 6 and 12 weeks (Figures 7A–F). Among such bacterial taxa, only *Comamonadaceae* showed a significant decrease among probiotic group subjects at 6 weeks compared to baseline levels (Figure 7D, *P* < 0.05). In contrast, *Bifidobacterium, Lactobacillus*, and *Prevotella 9* were increased from baseline at 12 weeks in the probiotics group (Figures 7G–I). At the family level, we found similar trends in both groups (Supplementary Figure 2).

**Figure 8:**
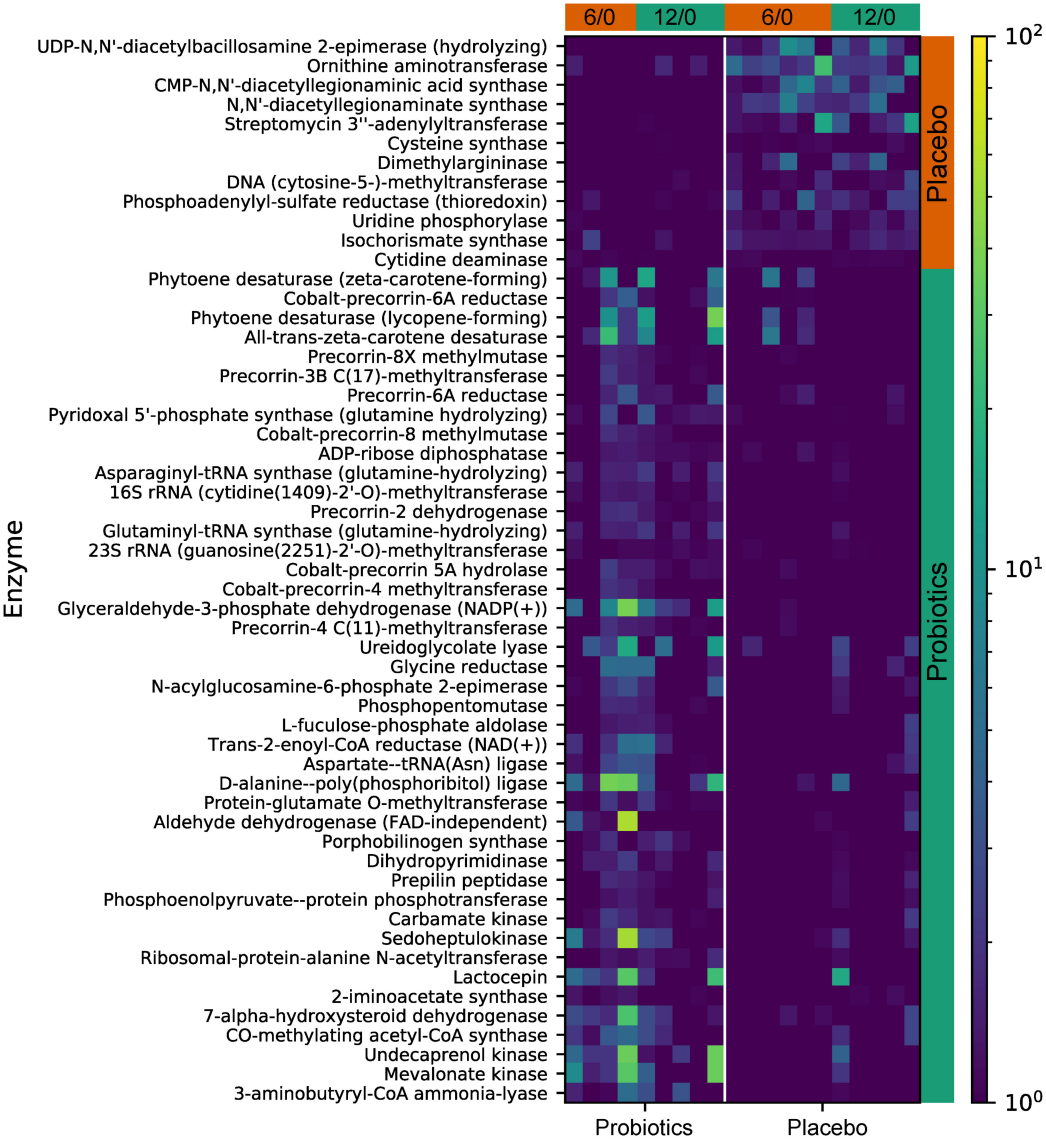
The predicted KEGG enzyme abundance based on PICRUSt2 functional gene analysis for the probiotics and placebo groups. The average abundance of KEGG enzyme differentially enriched in placebo and probiotics according to level 3.

Functional gene predictive analysis indicated that several genes had different abundances in the probiotics group after the 12-week treatment period. Notably, genes encoding the ubiquinone biosynthesis protein (ubiB, k03688), phytoene desaturase (EC:1.3.99.29), phytoene desaturase (lycopene-forming) (EC:1.3.99.31), and all-trans-zeta-carotene desaturase (EC:1.3.99.26) were all upregulated, while the genes encoding dimethylargininase (k01482) and acid phosphatase (phoN, k09474, EC:3.1.3.2) were downregulated (Figure 8). These findings do not meet the false discovery criteria for significance with multiple comparisons. The analysis results from the predicted KEGG pathway, shown in Supplementary Figure 3, and the predicted KO, shown in Supplementary Figure 4, further compare the gene expression of the probiotics and placebo groups.

### Correlation Between Gut Microbiota Abundances and Clinical Indices

**Figure 9:**
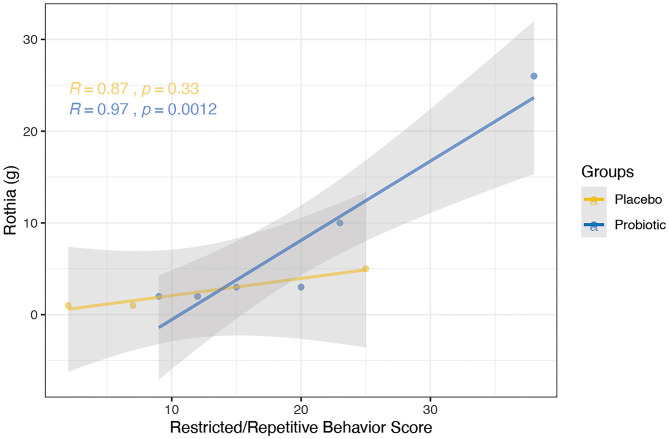
Correlation between the abundance of bacterial genera and clinical indices using spearman's method was performed for the probiotics (blue) and placebo (yellow) group at the 6-week time point. Probiotic group showed positive correlation between RRB scores and *Rothia* (*R* = 0.97, *P* < 0.005). No significant correlation was observed in the placebo group.

Clinical indices were correlated with the abundance of bacterial genera; one correlation was found to be significant in the probiotics group while no significant correlations were observed in the placebo group. Specifically, a positive correlation was discovered between the RRB scores and *Rothia* in the probiotics group at week 6 (Figure 9, *R* = 0.97, *P* < 0.005).

## Discussion

In our 12-week, randomized, double-blind, placebo-controlled trial of 68 PWS patients, BL-11 increased height in PWS subjects without changing weight. We observed that, during the treatment period, the probiotics group had a significantly greater height increase than the placebo group (*P* < 0.05). Interventions with other probiotics in the past have failed to elicit improvement in height (33). This study provides novel evidence for the use of BL-11 as an early intervention for patients with PWS. Interventions that lead to increased height in PWS may benefit patients at early developmental stages most and substantially improve long-term prognosis. PWS individuals were found to have absolute or functional Growth Hormone (GH) deficiency, and GH replacement is currently the most effective treatment for PWS (34, 35). GH was found not only to increase height, but also decrease body fat and improve cognition, motor, and mental function. With earlier initiation of GH treatment, better efficacy and prognostic benefit have been observed (35). One study found probiotics *L. reuteri* could increase growth hormone level in mice (36), which reveals a potential mechanism by which probiotics can enhance height and treat PWS patients: promotion of endogenous growth hormone release. Our findings warrant further investigation into the biological mechanisms of probiotics, a promising intervention for PWS with better tolerance and convenience than GH replacement (33).

We did not observe significant weight reduction within the intervention period, possibly due to the majority of our participants being < 5 years old, an age range at which obesity is not yet a major problem. Interestingly, the microbiome composition changes we observed with the intervention of *B. lactis* have been previously linked to weight or adiposity reduction (14–19, 22), improve fasting insulin sensitivity (22) and inflammatory attenuation (20, 21). Notably, we found a significant separation of the gut microbiome β-diversity between the probiotics and the placebo group after treatment. Baseline β-diversity has been directly correlated with long-term weight loss when adhering to a controlled diet (37). Therefore, probiotics supplementation may have preventative effects or may facilitate diet-induced weight reduction.

After administration of BL-11, we also noted reduction in the abundance of several bacterial genera and species that have been implicated in the pathology of obesity and associated inflammation. *Ruminococcaceae UCG-003*, associated with VLDL and metabolic syndrome, has also been implicated in inflammatory bowel diseases (38, 39). *Lachnospiraceae* ND3007 has been linked to elevated cholesterol, signs of insulin resistance, and infant obesity (40–42). Elevated *Streptococcus* has been associated with inflammatory GI disorders, maternal inflammation, bacteremia, and antibiotic use during pregnancy (43, 44). *Rothia* was found to have a higher abundance in a gestational diabetic cohort than a healthy pregnant cohort (45). The *Comamonadaceae* family is generally regarded as pathogenic in humans (46).

Conversely, *Bifidobacterium, Lactobacillus*, and *Prevotella* were each found to be considerably increased in the gut after BL-11 treatment. *Bifidobacterium*, the genus to which the interventional probiotic belongs, is widely regarded as beneficial to gut health and weight reduction (14–18, 20, 47–51). *Lactobacillus*, in addition to having protective effects against weight gain in humans, has been found to inhibit the activity of proinflammatory interleukins, which have been linked to obesity and poor obesity-related outcomes (52–54). The effect of *Prevotella* in the gut microbiome remains uncertain, as evidence linking this genus to health benefit and disease have both been reported. Wang et al. (55) reported that Prevotella-9 was found to be significantly decreased in both mice put on a high-fat diet and Zeng et al. (56) reported the same in women with PCOS who were insulin-resistant (55, 56). Further, Park et al. (57) reported an increased abundance of *Prevotella* in obesity improved mice and Kovatcheva-Datchary et al. (58) reported dietary fiber-induced improvements in post-prandial blood glucose and insulin were found to be positively associated with the abundance of *Prevotella* (57, 58). On the other hand, one study found that the Prevotellaceae family had greater relative abundance in 3 obese patients, compared to 3 normal weight patients (59). Another study, investigating fecal bacteria composition in HIV-positive patients, found that *Prevotella* was positively correlated with BMI, although most participants in this study had a BMI within the normal range (60). The conflicting findings about *Prevotella* in gut health and obesity may indicate the importance of balancing the abundance of this genus within the microbiome.

Furthermore, by using predictive functional gene analysis, we found the enhancement of antioxidant production-related pathways that exert anti-inflammatory and anti-obesity effects. The gene encoding the ubiquinone biosynthesis protein (ubiB, k03688), responsible for the biosynthesis of ubiquinone (CoQ10), was found to have increased abundance following probiotics treatment. CoQ10 supplementation can be useful in the treatment of a variety of chronic cardiovascular, inflammatory, and obesity-related disease (61). We also found an elevated abundance of genes encoding phytoene desaturase (EC:1.3.99.29), phytoene desaturase (lycopene-forming), (EC:1.3.99.31), and all-trans-zeta-carotene desaturase (EC:1.3.99.26), which all contribute to the biosynthesis of carotenoids, previously found to have beneficial effects on obesity and obesity-associated pathologies (62–64). We also found downregulation of two enzymes, dimethylargininase (k01482) and acid phosphatase (phoN, k09474, EC:3.1.3.2), which have been linked to obesity development and elevated cholesterol and triglyceride levels in human patients (65–67).

Taken together, the microbiome composition data and predictive functional gene analysis indicate that the diversity separation caused by BL-11 probiotics treatment favors protection against obesity and obesity-related pathology.

Although we did not find a significant change in psychological measurements (ASQ-3, ABC, SRS, and RRB), CGI-I showed significant overall improvement in the probiotics group after the treatment period compared with the placebo group (*P* < 0.05).

Interestingly, we found that the RRB score was positively correlated with *Rothia* at the genus level (*P* < 0.005). RRB is one of the core symptoms of ASD, which has been reported in as many as 25–40% of PWS cases (3, 68). *Rothia*, in addition to being linked to diabetes (45), has been reported to be more prevalent in children with ASD than typically-developing children (12.2-fold-change; FDR, *P* < 0.05) (69). While the mechanism by which the BL-11 improved clinical impression of PWS patients is unknown, both the correlation found between *Rothia* and RRB and alterations in the gut microbiome composition may be associated with the administration of BL-11. Such findings may further implicate modulatory effects on brain functions or host metabolism via signaling through the gut-brain axis. Further investigation of *Rothia* and other microbiome markers may reveal potent and feasible targets for neuropsychiatric therapies.

Our randomized trial showed that treatment with probiotic *B. Lactis* strain (BL-11) for 12 weeks significantly increased height, a novel finding with vital implications for early treatment in PWS. Probiotic treatment may improve overall psychological clinical symptoms, as suggested by CGI-I results. In conjunction with its observed effects in inducing favorable alterations in the gut microbiome composition and functional profile, our results suggest that *B. lactis* is a viable probiotic candidate for facilitating the improvement in obesity-related gut microbiome dysbiosis in individuals with PWS, thereby potentially inducing a reduction of obesity in such populations. There are some limitations to the study that deserve consideration. First, despite our adoption of proper subject recruitment procedures, subject retention strategies, and data collection plans, the enrollment, retention, and data collection of PWS participants were challenging, thus leading to reduced sample sizes as anticipated and missing data, which limited further subgroup analysis. Second, although there was no statistical difference in clinical indices between the probiotics and placebo groups at baseline, the broad age range of enrolled participants in this study resulted in high subject population heterogeneity and may contribute to the variability of the treatment efficacy. Third, assessment of fecal microbiome was not controlled for dietary habits, which may influence the microbial abundances at the individual level. Thus, future studies with larger sample sizes, improved control for environmental factors, and subgroup stratification are warranted. Due to the limitations of the study listed above, further studies are warranted to investigate the mechanism and efficacy of BL-11 probiotics treatment in PWS.

## Data Availability Statement

The datasets presented in this study can be found in online repositories. The names of the repository/repositories and accession number(s) can be found at: https://www.ncbi.nlm.nih.gov/bioproject/PRJNA643297.

## Ethics Statement

The studies involving human participants were reviewed and approved by Internal Review Board (IRB) of the Second Affiliated Hospital of Kunming Medical University. Written informed consent to participate in this study was provided by the participants' legal guardian/next of kin.

## Author Contributions

X-JK conceived the concept, developed experimental design and trial protocol, provided resources and major funding, led the trial conduction, finished the first draft, and major revisions of the manuscript. XL, XZ, RT, SL, JZ, JW, YW, ZY, CS, XCu, TD, MF, and HC contributed on subject interview and data collection. XCa and GW helped admin works. RT and KL contributed on data analysis and creation of figures and tables while JL provided assistance. KL, CC, BW, HS, and AL contributed writing and editing. All authors have read and agreed to the published version of the manuscript.

## Conflict of Interest

The authors declare that the research was conducted in the absence of any commercial or financial relationships that could be construed as a potential conflict of interest.
