# Supplementary material for: The Effects of Probiotic Supplementation on Anthropometric Growth and Gut Microbiota Composition in Patients With Prader-Willi Syndrome: A Randomized Double-Blinded Placebo-Controlled Trial
Source: Front Nutr. 2021 Feb 19;8:587974. doi: 10.3389/fnut.2021.587974 (PMC7933553; doi:10.3389/fnut.2021.587974)
Supplement: Supplementary file 1 [file Data_Sheet_2.DOCX]

Supplementary Material

**Supplementary Figures and Tables**

**Supplementary Table S1.** The reasons for drop out and co-morbid symptoms of study participants.

|  | **Probiotic** | **Placebo** | ***P-*value*** |
| --- | --- | --- | --- |
| **Co-morbid Symptoms** |  |  |  |
| GI Symptoms (n = 41, 47.5% has ≥ 1) | 0.87 ± 1.63 | 1.39 ± 1.75 | 0.24 |
| Allergies (n = 41, 78.5% has ≥ 1) | 1.78 ± 1.59 | 1.83 ± 1.25 | 0.74 |
| RRB (n = 41, 100% ≥ 1) | 19.2 ± 10.3 | 16.7 ± 6.61 | 0.56 |
| Sleep Apnea (n = 65) | 27% | 20% | 0.48 |
| Scoliosis (n = 65) | 33% | 17% | 0.12 |
| Undescended Testicle (n = 65) | 58% | 51% | 0.61 |
| **Reasons for Drop out** |  |  |  |
| Self-withdraw (n = 68) | 24% | 24% | 1 |
| Between 0-6 weeks | 5 | 6 | 1 |
| Between 6-12 weeks | 3 | 2 |  |
| Antibiotic use (n = 68) | 5 | 3 | 0.48 |
| Between 0-6 weeks | 3 | 0 | 0.35 |
| Between 6-12 weeks | 2 | 3 |  |
| Adverse events (n = 68) | 0% | 0% | 1 |
| Between 0-6 weeks | 0% | 0% | 1 |
| Between 6-12 weeks | 0% | 0% | 1 |

* Chi-square test and Wilcoxon sum rank tests were used to examine the difference between probiotic and placebo groups, p >0.05 in all listed co-morbid symptoms.


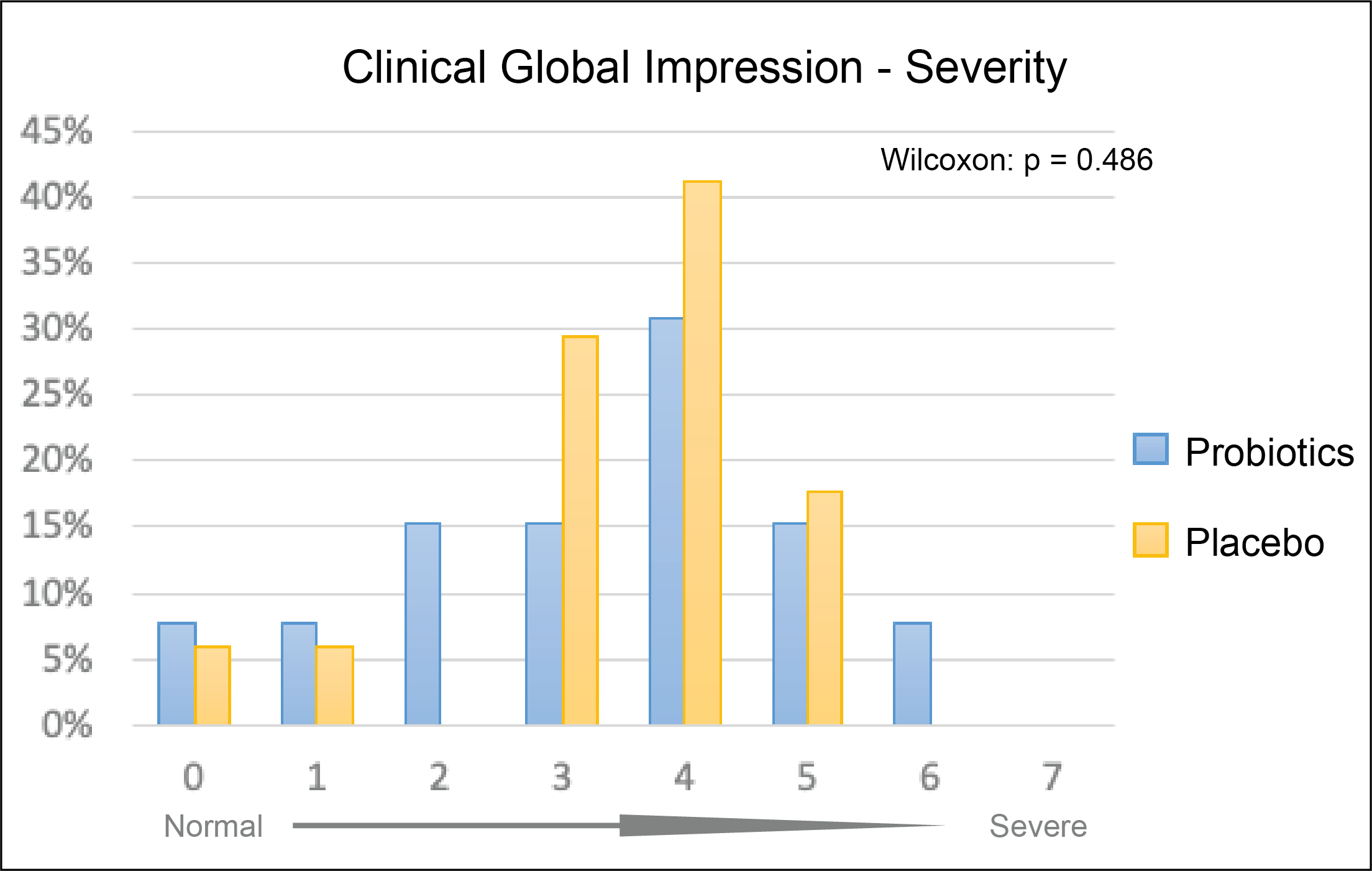


**Supplementary Figure S1.** Clinical Global Impression (CGI) – Severity at Baseline between two groups. Comparison of CGI-S at baseline between probiotics group (blue) and placebo group (yellow). There is no difference in overall severity level found between groups (p > 0.05).


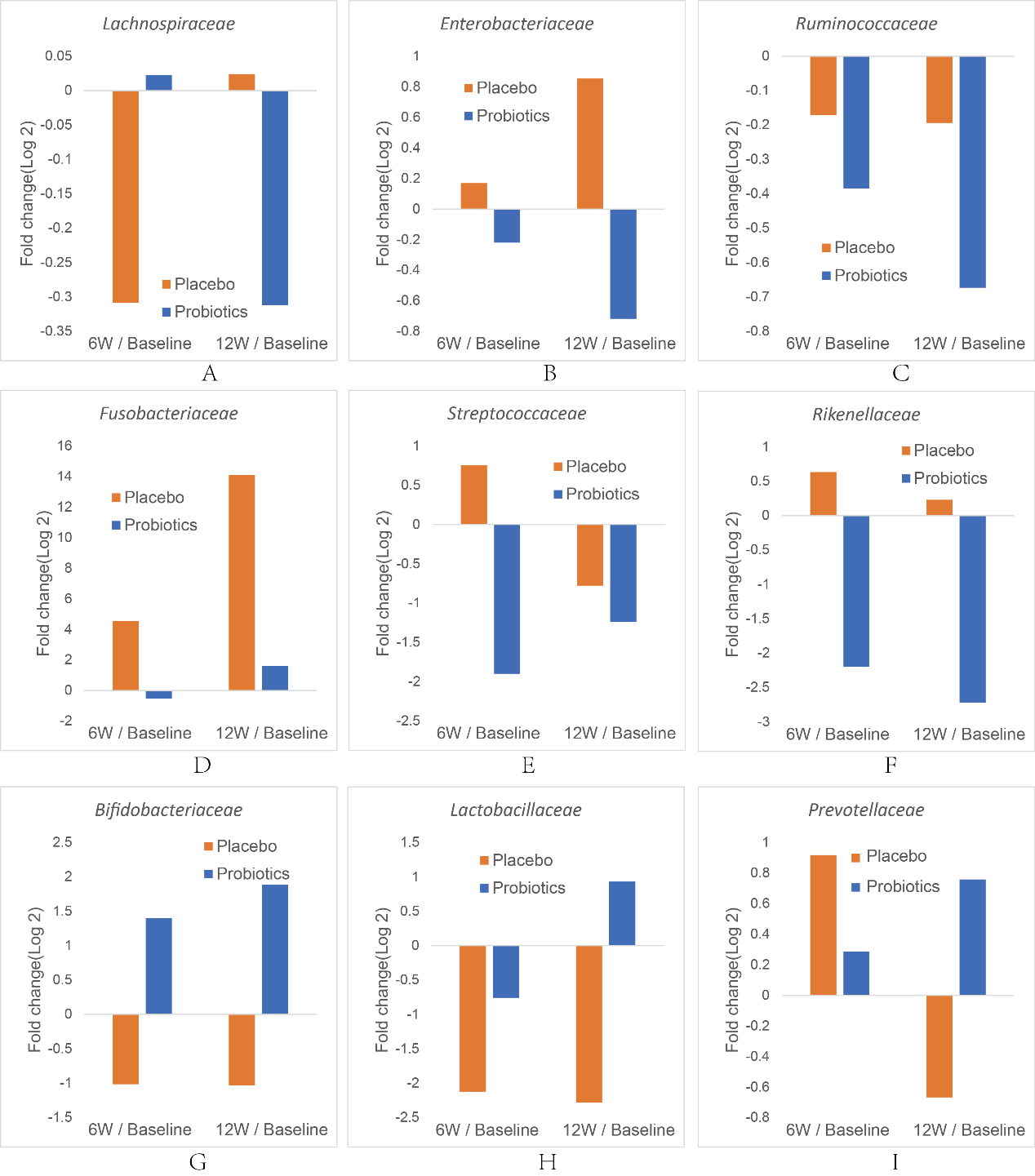


**Supplementary Figure S2.** Fold change of relative of abundance at family level. Each bar represents the log 2 transferred relative change of gut microbial abundance compared with the baseline at 6 and 12 weeks.


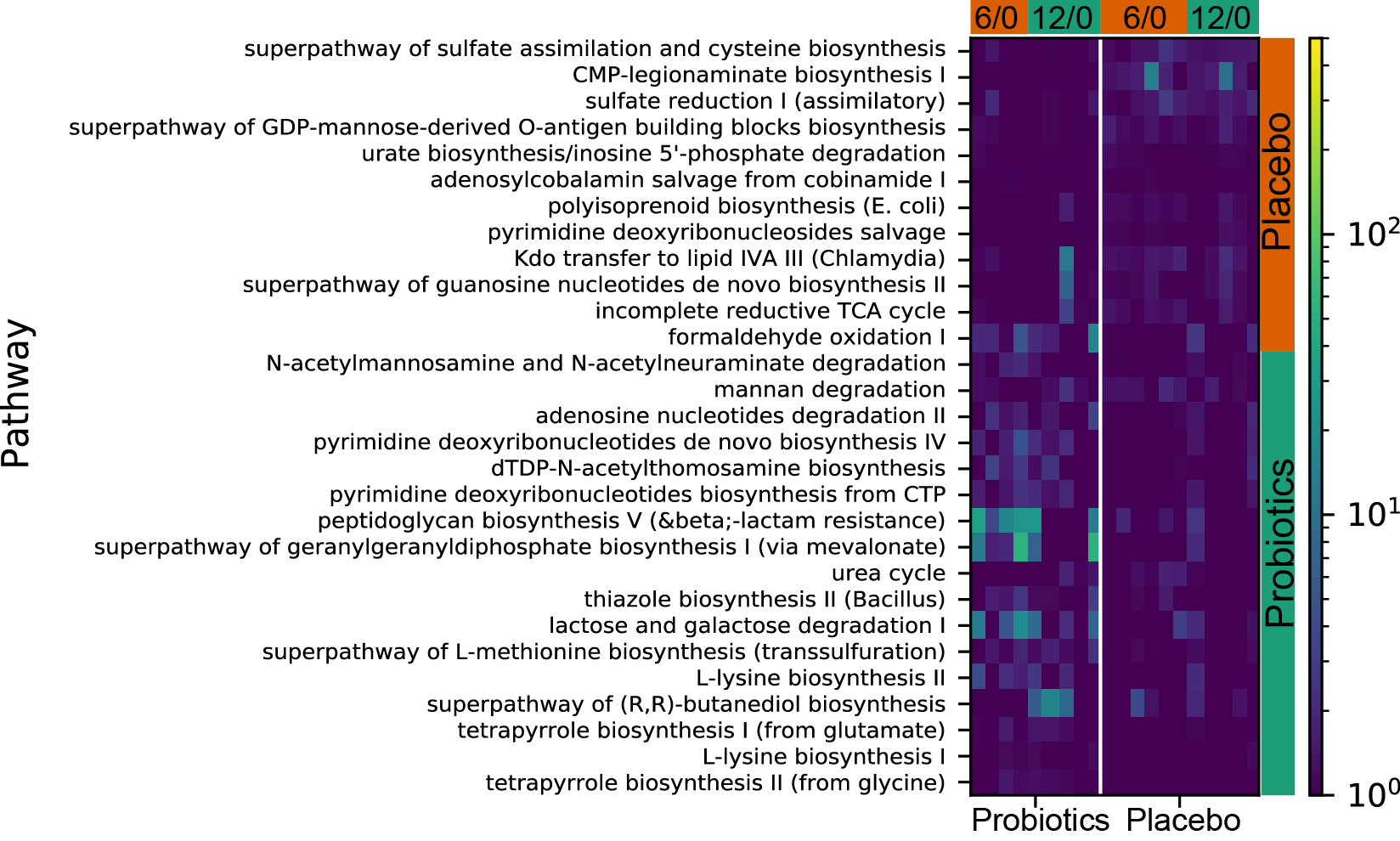


**Supplementary Figure S3.** Comparison of the predicted KEGG pathway in placebo and probiotics groups. The average abundance of KEGG pathway differentially enriched in placebo and probiotics according to level 1.

**
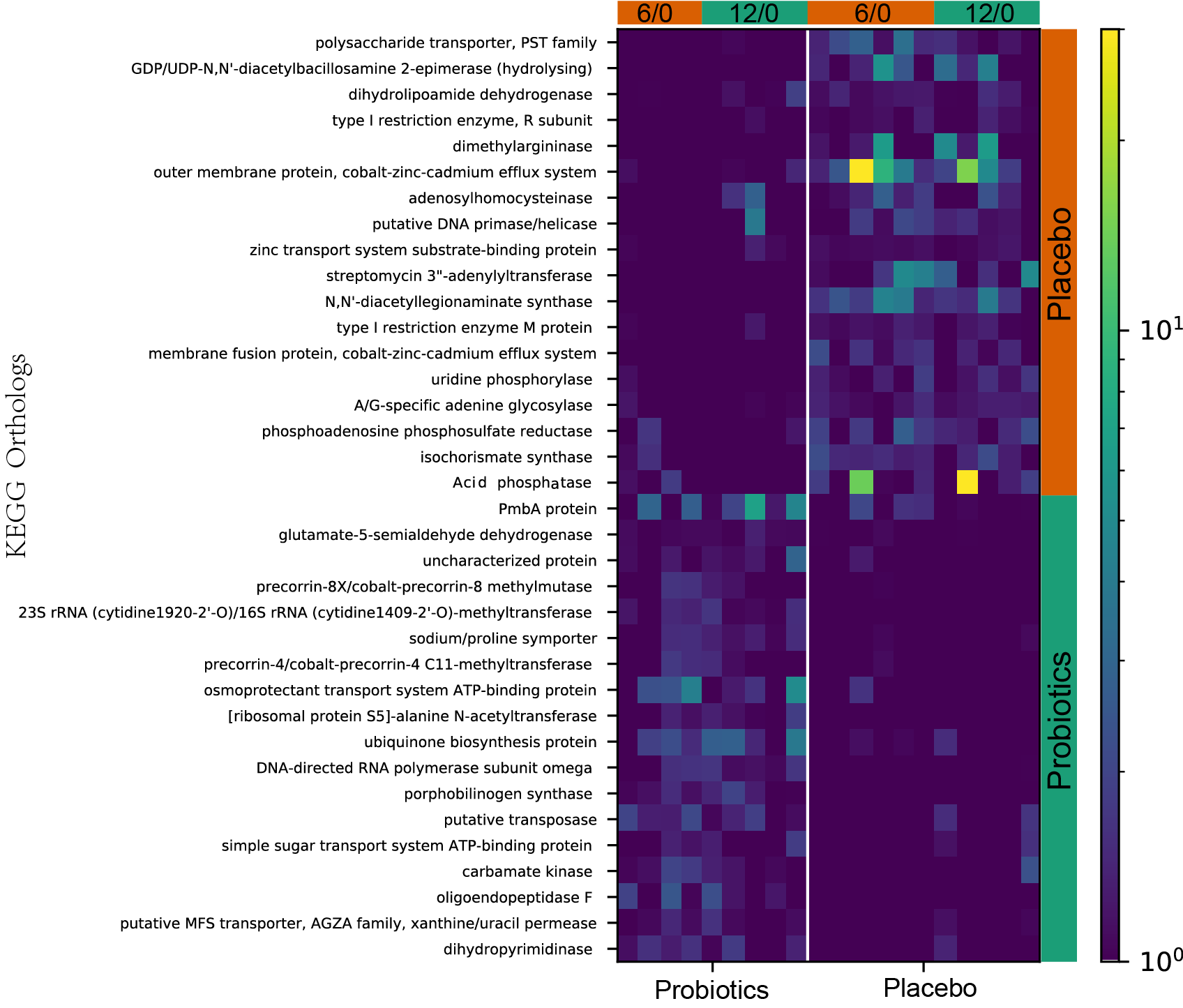
**

**Supplementary Figure S4.** Comparison of the predicted KEGG orthologous (KO) between placebo and probiotics groups. The average abundance of KEGG pathway differentially enriched in placebo and probiotics according to level 2.
